# Heat- and Cold-Related Mortality Burden in the US From 2000 to 2020

**DOI:** 10.1001/jamanetworkopen.2025.42269

**Published:** 2025-11-07

**Authors:** Lingzhi Chu, Robert Dubrow, Kai Chen

**Affiliations:** 1 Department of Environmental Health Sciences, Yale School of Public Health, New Haven, Connecticut; 2 Yale Center on Climate Change and Health, Yale School of Public Health, New Haven, Connecticut

## Abstract

**Importance:**

With a warming climate, it is essential to have up-to-date and spatiotemporally resolved quantification of the national mortality burden attributable to nonoptimal temperatures.

**Objective:**

To investigate the mortality burden associated with heat and cold in the entire contiguous US from 2000 to 2020.

**Design, Setting, and Participants:**

This case series assessed death records from the National Center for Health Statistics for all counties within the contiguous US from January 1, 2000, to December 31, 2020. The study used 2-stage modeling, with the first stage being a case-crossover design with county-specific conditional logistic regression of mortality on daily mean temperature incorporating distributed lag nonlinear models and the second stage pooling the estimates by metaregression. Based on the estimated exposure-response functions for temperature and mortality, attribution analyses for low and high temperatures were performed. Calendar time trends were analyzed, and stratified analyses by cause of death and demographic factors (ie, age, sex, and marital status) were performed. This analysis was conducted from August 9, 2024, to June 16, 2025.

**Exposures:**

Daily mean temperature and dewpoint temperature at a 4-km resolution for the contiguous US from the Parameter-Elevation Relationships on Independent Slopes Model (PRISM) climate database.

**Main Outcomes and Measures:**

All-cause mortality and 7 specific causes, including endocrine, nutritional and metabolic diseases; mental, behavioral and neurodevelopmental disorders; nervous system diseases; circulatory diseases; respiratory diseases; digestive diseases; and external causes.

**Results:**

A total of 54 223 429 deceased individuals were included in the analyses. Most decedents were 65 years or older (73.4%); 50.2% were male, 37.4% were married, and 35.1% were widowed. Both low and high temperatures were associated with increased odds of death within 0 to 6 days. Compared with the minimum mortality temperature, temperatures at the 5th and 95th percentiles were associated with odds ratios (ORs) of 1.057 (95% CI, 1.051-1.064) and 1.011 (95% CI, 1.009-1.013), respectively. The excess annual deaths attributable to low and high temperatures were estimated to be 45 992 (95% CI, 28 639-63 202) and 3414 (95% CI, 1650-5173), respectively, with regional variations and an increase in the high temperature burden from 2670 annual deaths during the 2000-2009 period to 4091 during the 2010-2020 period. Heterogeneity existed by cause of death, age, sex, and marital status.

**Conclusions and Relevance:**

In this case series, nonoptimal temperatures were critical environmental contributors associated with mortality burden, with differential vulnerability by geographic location, cause of death, and demographic factors. These findings highlight the need for urgent actions against temperature-related health burdens through tailored climate and public health strategies, considering the local context and demographic profiles.

## Key Points

**Question:**

From 2000 to 2020 in the contiguous US, what were the spatiotemporal patterns in the mortality burden from nonoptimal temperatures?

**Findings:**

This case series of 54 223 429 deceased individuals found that both low and high temperatures were significantly associated with mortality burden, with low temperatures associated with more mean annual deaths (45 992) than high temperatures (3414). However, the burden from high temperatures increased by 53% from the 2000-2009 to 2010-2020 study periods; vulnerabilities varied by region, cause of death, and demographic factors (age, sex, and marital status).

**Meaning:**

These findings suggest that tailored public health and climate strategies, especially those incorporating local contexts and demographic differences, are needed to mitigate temperature-related risks and protect vulnerable populations.

## Teaser

This case series evaluates the all-cause and cause-specific mortality burden attributable to nonoptimal temperatures in the US using recent complete death records.

## Introduction

The world experienced its record-breaking warmest year in 2024 along with unusually low temperatures in the US in the winter of 2025.^1,2^ Consequently, health impacts of nonoptimal temperatures are increasing. Statistics from the Centers for Disease Control and Prevention have revealed increasing cold- and heat-related mortality rates in recent years in the US.^3,4,5,6^ However, these statistics restricted cold- and heat-related deaths to individuals with an underlying or contributing cause directly related to cold or heat exposure (eg, hypothermia, exposure to excessive natural heat), so likely missed deaths from other causes (eg, cardiovascular, respiratory, renal) that were triggered by nonoptimal temperatures, but in which cold or heat played a less evident role, making their attribution as underlying or contributing causes of death less likely.^7,8^

Research on temperature-related mortality burden in the US has used the Global Burden of Disease (GBD) database,^9,10^ the Multi-Country Multi-City (MCC) Collaborative Research Network database,^11,12,13,14,15^ and/or US national databases.^10,16,17^ However, these studies are limited in providing up-to-date, comprehensive, stratified estimates with truly national coverage. First, the health impacts of ambient temperature may change over calendar time, especially in recent decades with a rapidly warming climate, while some studies were based on old data (eg, 1980-1988 in the GBD studies or 1995-2009 in the MCC studies). Second, most studies did not have full national coverage, but only included mortality data from major metropolitan areas or cities and neglected nonurban areas that may also face significant mortality risks from nonoptimal temperatures. Third, cause-specific attribution analyses were rarely conducted. Last, temperature-attributable mortality may be heterogeneous by demographic characteristics (eg, age, sex). The present study aimed to evaluate the all-cause and cause-specific mortality burden attributable to nonoptimal temperatures using recent complete death records in the US, to investigate the spatiotemporal patterns in temperature-related mortality burden, and to assess heterogeneity by age, sex, and marital status.

## Methods

The Yale University Institutional Review Board approved this case series; the need for informed consent was waived because the study was deemed non–human participant research. The study followed the Appropriate Use and Reporting of Uncontrolled Case Series in the Medical Literature reporting guideline.

### Main Outcomes

We obtained complete death records from the National Center for Health Statistics from January 1, 2000, to December 31, 2020, covering all 3108 counties in the contiguous US according to the 2020 Census Cartographic Boundary of counties. The records included individual-level demographic information (ie, age, sex, and marital status), date and county of death occurrence, and the underlying cause of death coded according to the *International Statistical Classification of Diseases, Tenth Revision* (*ICD-10*).

We evaluated all-cause mortality and 7 specific causes: endocrine, nutritional, and metabolic diseases (*ICD-10* codes E00-E89); mental, behavioral, and neurodevelopmental disorders (*ICD-10* codes F01-F99); nervous system diseases (*ICD-10* codes G00-G99); circulatory diseases (*ICD-10* codes I00-I99); respiratory diseases (*ICD-10* codes J00-J99); digestive diseases (*ICD-10* codes K00-K95); and external causes (*ICD-10* codes V00-Y99). Other causes were not evaluated per our data use agreement.

### Exposure Assessment

We extracted daily mean temperature and dewpoint temperature at a 4-km resolution for the contiguous US from the Parameter-Elevation Relationships on Independent Slopes Model (PRISM) climate database (2000-2020).^18^ We then calculated the county-level daily mean temperature and dewpoint temperature based on the PRISM data, weighted by annual population at a 1-km resolution from the LandScan global population database.^19^ We also calculated county-level population-weighted daily mean fine particulate matter (PM_2.5_) concentrations (2000-2020) based on daily 1-km gridded PM_2.5_ estimates from the USHighAirPollutants dataset and the LandScan global population data.^20^

### Census Sociodemographic Data and Rural-Urban Classifications

We extracted county-level census statistics from American Community Survey data, including population, percentage of Black population, percentage of Hispanic population, percentage of older population (aged ≥65 years), percentage of population with a high school diploma or higher level of education among individuals 25 years or older, and median household income. Race and ethnicity data were collected for the second stage of our statistical analysis (ie, metaregression) to help explain the potential heterogeneity across counties. We calculated means of the American Community Survey 5-year summary data in 2010 (covering 2006-2010) and 2015 (covering 2011-2015) to represent the average conditions during the overall study period (2000-2020). We also obtained the US Department of Agriculture’s 2013 Rural-Urban Continuum Codes, which classify counties into metropolitan vs nonmetropolitan areas with 9 refined groups.^21^

### Statistical Analysis

Analyses were conducted from August 9, 2024, to June 16, 2025. Considering the differential temperature distributions across geographic locations, we evaluated the relationship between temperature and mortality using a 2-stage design with county-specific analyses at the first stage and metaregression at the second stage.^22^

In the first stage, we used a time-stratified case-crossover design in which each case (ie, death) served as its own control, with the date of death serving as the case date and the dates with the same day of week within the same calendar month and year serving as control dates. This design automatically controlled for day of week, seasonality, long-term trends, and individual-level time-invariant characteristics.

For each county, we fitted a distributed lag nonlinear model combined with conditional logistic regression at a lag of 0 to 6 days, using natural cubic splines for both the variable (knots: county-specific 10th, 75th, and 90th percentiles of temperature) and the lag (equally placed knots with *df* = 3) dimensions for temperature. We controlled for dewpoint temperature (equally placed knots for both the variable [*df* = 4] and the lag [*df* = 3] dimensions).

In the second stage, we conducted multivariate random-effects metaregression by regressing the county-specific coefficient estimates from the first stage on county-level climate characteristics (ie, mean temperature and temperature range from 2000 to 2020) and census sociodemographic characteristics (ie, population; percentages of Black population, Hispanic population, older population, and high school graduate or higher educational attainment; and median household income). In our preliminary analyses including all these variables at the second stage, we found that population and percentage of older population were not statistically significant in Wald tests, and these 2 variables were thus excluded in the main analysis. We then obtained the pooled exposure-response curves for the contiguous US using the population-weighted climate and sociodemographic characteristic values over counties in the fitted metaregression model; we obtained county-specific exposure-response curves using county-specific values.

For each county, we calculated the minimum mortality temperature (MMT; restricted between the 1st and 99th percentiles of temperature) and then estimated the mortality burden (ie, number of deaths) attributable to cold (temperatures below the MMT) and heat (temperatures above the MMT). Basically, we first estimated the attributable fraction (AF) associated with respective days based on the exposure-response curve, assuming the estimated odds ratio (OR) approximated the relative risk, and then calculated attributable cold- or heat-related death counts (AF × observed daily death count) on respective days and summed across days.

We conducted stratified analyses by individual-level cause of death, age (<5, 5-24, 25-44, 45-64, 65-74, 75-84, or >84 years), sex (female or male), and marital status (single, married, divorced, or widowed), county-level census sociodemographic characteristics in tertiles, and urbanicity (metropolitan vs nonmetropolitan counties). For stratified analyses by individual characteristics, to address the issue of small sample sizes in many counties, we only included counties with at least 500 deaths when fitting metaregression models at the second stage, but all counties were included in the attribution analysis. We used *Q* statistics to test for heterogeneity by age, sex, and marital status.

We performed sensitivity analyses to validate the robustness of our primary estimates: (1) varying the time window to a lag of 0 to 3, 0 to 13, and 0 to 20 days; (2) varying knots for the temperature variable dimension to county-specific quartiles; (3) additionally controlling for moving averages of daily mean PM_2.5_ concentration over a lag of 0 to 6 days (specified as linear); (4) using daily maximum or minimum temperature as the primary exposure metric; and (5) in stratified analyses, requiring counties to have at least 200 or at least 1000 deaths to be included in metaregression models. We performed all analyses in R, version 4.4.2 (R Program for Statistical Computing) and considered a 2-sided *P* < .05 as statistically significant.

## Results

We included 54 223 429 deceased individuals in the contiguous US from 2000 to 2020. Among these, 17 770 295 deaths (32.8%) were due to circulatory diseases and 5 207 655 (9.6%) were due to respiratory diseases (Table). Most deaths occurred among individuals 65 years or older (65-74 years, 9 552 014 [17.6%]; 75-84 years, 13 973 052 [25.8%]; and >84 years, 16 256 116 [30.0%]), with 10 332 336 (19.1%) among those aged 45 to 64 years. The decedents were almost evenly divided between female (27 005 834 [49.8%]) and male (27 217 595 [50.2%]) individuals; 20 296 160 (37.4%) were married and 19 043 047 (35.1%) were widowed.

**Table.** Study Population in the Contiguous US (2000-2020)

Characteristic	No. (%) (N = 54 223 429)
Cause of death (*ICD-10* codes)	
Endocrine, nutritional, and metabolic diseases (E00-E89)	2 317 417 (4.3)
Mental, behavioral, and neurodevelopmental disorders (F01-F99)	2 259 497 (4.2)
Nervous system diseases (G00-G99)	3 220 052 (5.9)
Circulatory diseases (I00-I99)	17 770 295 (32.8)
Respiratory diseases (J00-J99)	5 207 655 (9.6)
Digestive diseases (K00-K95)	2 012 879 (3.7)
External causes (V00-Y99)	4 153 924 (7.7)
Age, y	
<5	619 386 (1.1)
5-24	790 799 (1.5)
25-44	2 692 505 (5.0)
45-64	10 332 336 (19.1)
65-74	9 552 014 (17.6)
75-84	13 973 052 (25.8)
>84	16 256 116 (30.0)
Unknown	7221 (0.01)
Sex	
Female	27 005 834 (49.8)
Male	27 217 595 (50.2)
Marital status	
Single	6 862 344 (12.7)
Married	20 296 160 (37.4)
Divorced	7 569 505 (14.0)
Widowed	19 043 047 (35.1)
Unknown	452 373 (0.8)

Abbreviation: ICD-10, International Statistical Classification of Diseases, Tenth Revision.

### Temperature-Mortality Associations

**Figure 1.**
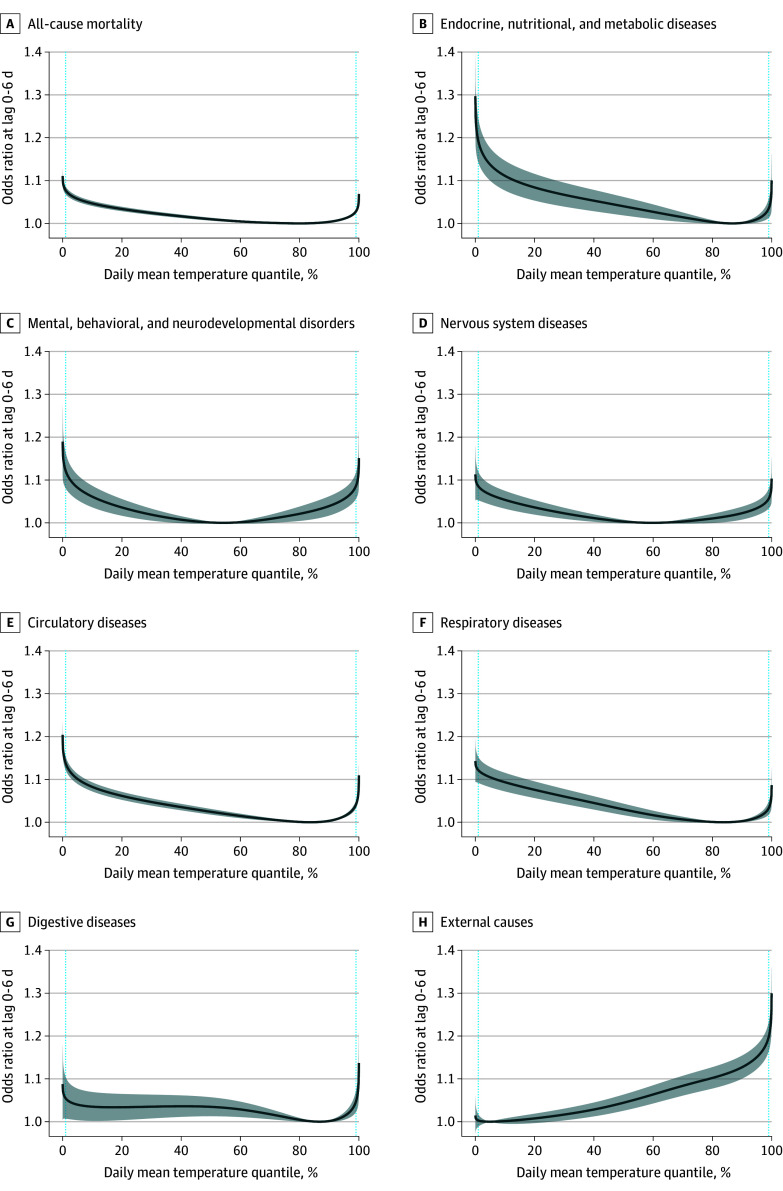
Associations Between Temperature and All-Cause and Cause-Specific Mortality Odds ratios (blue curves) with 95% CIs (shaded areas) are reported, with the respective minimum mortality temperature as the reference. The dotted, light blue vertical lines indicate the 1st and 99th percentiles of temperature. For analyses of specific causes of death, only counties with at least 500 deaths were included at the second stage of metaregression to address the convergence issue.

We observed significantly increased all-cause mortality associated with both low and high temperatures at lag 0 to 6 days (Figure 1). With MMT (approximately the 80th percentile) as the reference, temperatures at the 5th percentile had an OR of 1.057 (95% CI, 1.051-1.064); at the 10th percentile, 1.047 (95% CI, 1.041-1.053); at the 90th percentile, 1.004 (95% CI, 1.002-1.005); and at the 95th percentile, 1.011 (95% CI, 1.009-1.013) (eTable 1 in Supplement 1).

The association differed by cause of death (Figure 1). Most causes showed associations with both low and high temperatures, except for mortality due to external causes, which was only associated with high temperatures.

**Figure 2.**
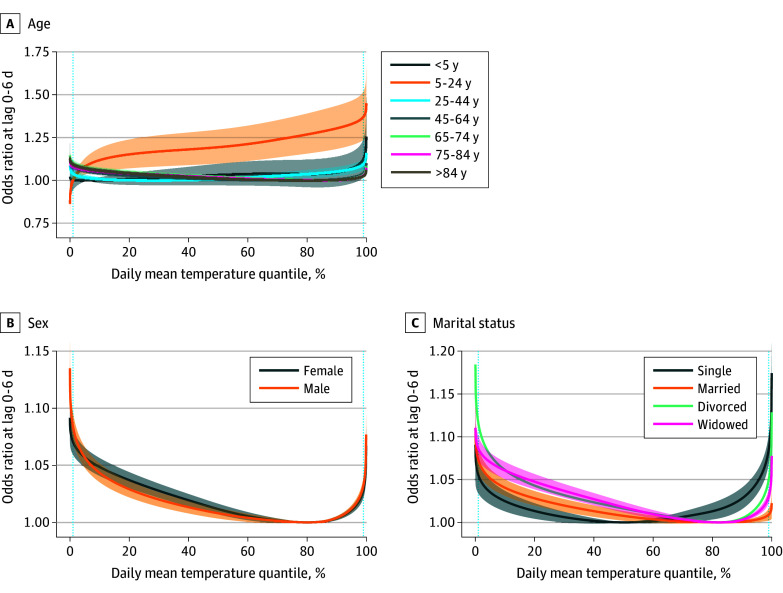
Associations Between Temperature and All-Cause Mortality Stratified by Age, Sex, and Marital Status Odds ratios with 95% CIs (shaded areas) are reported, with the respective minimum mortality temperature as the reference. The dotted, light blue vertical lines indicate the 1st and 99th percentiles of temperatures. Only counties with at least 500 deaths were included at the second stage of metaregression to address the convergence issue.

We observed significantly heterogenous associations between temperature and all-cause mortality by age, sex, and marital status (Figure 2 and eTable 2 in Supplement 1). Mortality among children younger than 5 years was associated only with extremely high temperatures (OR at the 99th percentile, 1.123 [95% CI, 1.018-1.239]). Mortality among adolescents and young adults aged 5 to 24 years was associated with high temperatures, but not low temperatures (OR at the 95th percentile, 1.329 [95% CI, 1.205-1.465]) (eTable 3 in Supplement 1). Mortality among adults older than 24 years was associated with both low and high temperatures, with the OR at low temperatures generally increasing with increasing age (OR at the 5th percentile, 1.018 [95% CI, 1.001-1.034] for individuals aged 25-44 years, 1.056 [95% CI, 1.041-1.071] for those aged 45-64 years, 1.074 [95% CI, 1.059-1.090] for those aged 65-74 years, 1.063 [95% CI, 1.051-1.075] for those aged 75-84 years, and 1.070 [95% CI, 1.058-1.082] for those older than 84 years). Females had higher ORs at low temperatures (OR, 1.049 [95% CI, 1.041-1.056] at the 10th percentile) compared with males (OR, 1.043 [95% CI, 1.035-1.052] at the 10th percentile). Divorced and widowed individuals had higher ORs at lower temperatures compared with single and married individuals (OR at the 5th percentile, 1.079 [95% CI, 1.061-1.096] for divorced individuals and 1.070 [95% CI, 1.059-1.081] for widowed individuals compared with 1.035 [95% CI, 1.021-1.050] for single individuals and 1.051 [95% CI, 1.041-1.062] for married individuals), while single individuals had higher ORs at higher temperatures (OR at the 95th percentile, 1.046 [95% CI, 1.033-1.060] compared with 1.003 [95% CI, 1.000-1.006] for married individuals, 1.018 [95% CI, 1.013-1.023] for divorced individuals, and 1.011 [95% CI, 1.008-1.014] for widowed individuals).

### Attributable Burden

Between 2000 and 2020 in the contiguous US, the AF for low temperatures and all-cause mortality was 1.78%, comprising a mean of 45 992 (95% CI, 28 639-63 202) excess annual deaths; the AF for high temperatures and all-cause mortality was 0.13%, comprising a mean of 3414 (95% CI, 1650-5173) excess annual deaths (eTable 4 in Supplement 1). To eliminate COVID-19 as a factor, we conducted an ad hoc analysis excluding 2020 and found a mean of 43 263 (95% CI, 25 988-60 394) excess annual deaths attributable to low temperatures and 3186 (95% CI, 1469-4894) excess annual deaths attributable to high temperatures.

**Figure 3.**
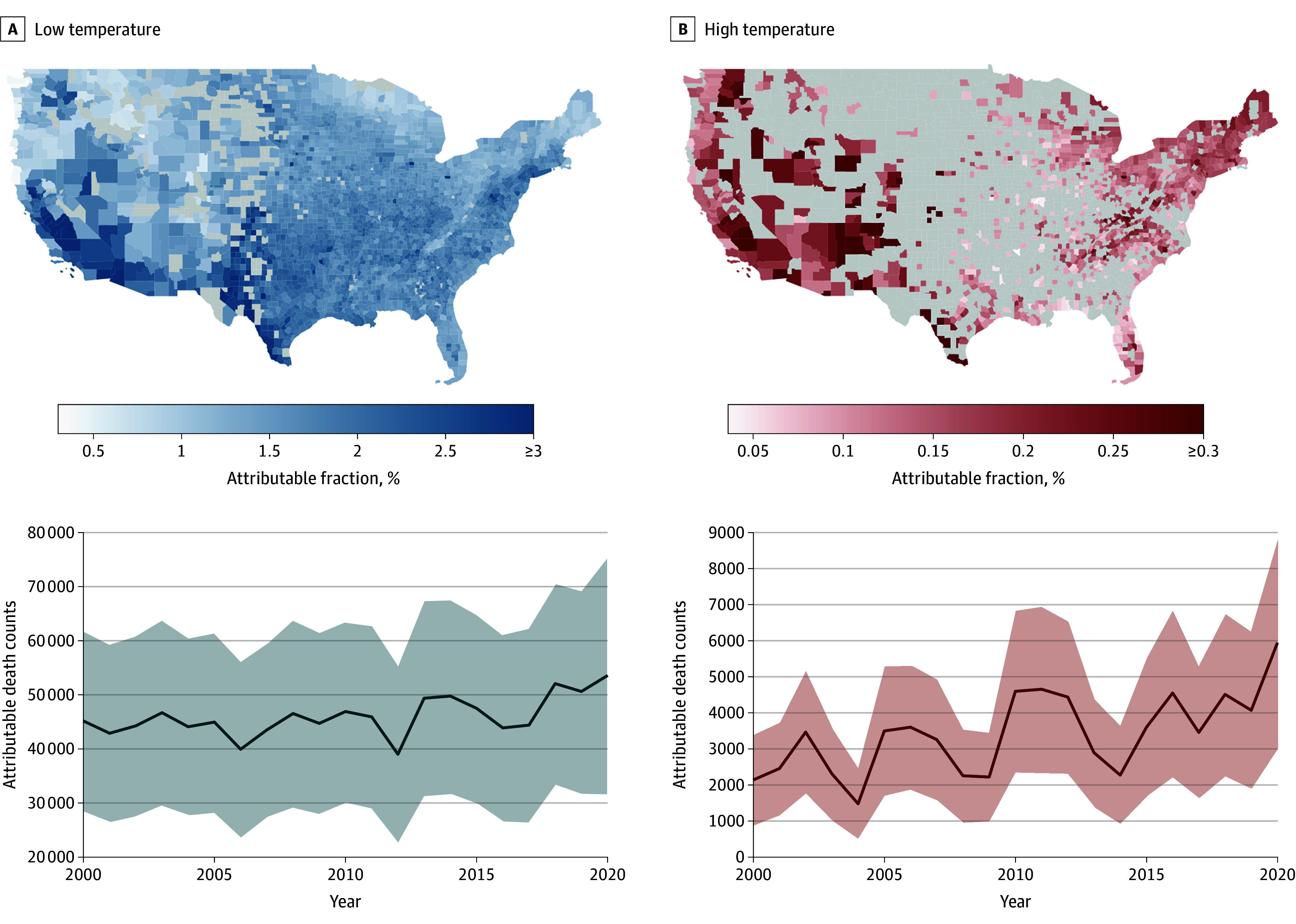
Spatial and Temporal Patterns of All-Cause Mortality Attributable to Low and High Temperatures Complying with data use policy, counties with fewer than 10 attributable deaths are shown as missing (indicated by gray) on the maps. However, the contribution of these counties was included in the attributable death counts and in statistical analyses. Shaded areas indicate 95% CIs.

We observed no obvious spatial pattern of excess annual mortality rates (eFigure 1 in Supplement 1). However, using AF as the indicator, in general the southwestern US had higher AFs due to low temperatures and the western US had higher AFs due to high temperatures, compared with other regions (Figure 3 and eFigure 2 in Supplement 1). We did not find strong evidence for heterogeneity across counties by county-level sociodemographic characteristics or urbanicity that might explain this spatial variation (eFigure 3 in Supplement 1).

The annual mortality count attributable to low temperatures increased by 7% between the 2000-2009 and 2010-2020 study periods, from 44 278 to 47 551 annual deaths. However, the annual mortality count attributable to high temperatures increased by 53%, from 2670 to 4091 annual deaths (Figure 3 and eTable 4 in Supplement 1).

**Figure 4.**
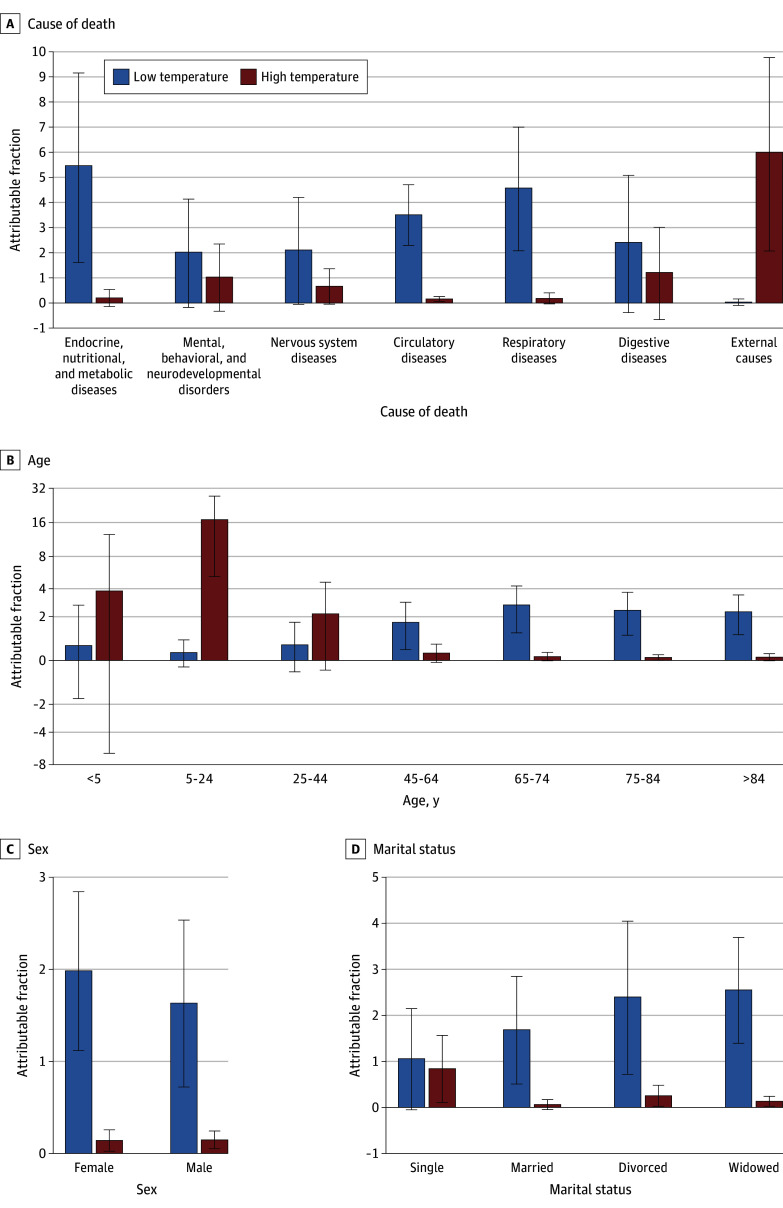
Attributable All-Cause Mortality Stratified by Cause of Death, Age, Sex, and Marital Status Error bars indicate 95% CIs.

We observed significant mortality fractions attributable to low temperatures for endocrine, nutritional, and metabolic disease (AF, 5.5% [95% CI, 1.6%-9.2%]), circulatory diseases (AF, 3.5% [95% CI, 2.3%-4.7%]), and respiratory diseases (AF, 4.6% [95% CI, 2.1%-7.0%]). Significant mortality fractions attributable to high temperatures were observed for circulatory diseases (AF, 0.2% [95% CI, 0.1%-0.3%]) and external causes (AF, 6.0% [95% CI, 2.1%-9.8%]) (Figure 4 and eTable 5 in Supplement 1).

The attributable mortality burden varied by demographic characteristics. Individuals 45 years or older had significant mortality fractions attributable to low temperatures (AFs, 1.7% [95% CI, 0.4%-2.9%] for 45-64 years of age; 2.7% [95% CI, 1.2%-4.3%] for 65-74 years of age; 2.4% [95% CI, 1.1%-3.7%] for 75-84 years of age; and 2.3% [95% CI, 1.1%-3.5%] for older than 84 years), while individuals aged 5 to 24 years (AF, 17.0% [95% CI, 5.2%-27.3%]) and 75 to 84 years (AF, 0.1% [95% CI, 0.01%-0.2%]) had significant mortality fractions attributable to high temperatures (Figure 4 and eTable 5 in Supplement 1). Females had a slightly higher AF than males due to low temperatures (2.0% [95% CI, 1.1%-2.8%] vs 1.6% [95% CI, 0.7%-2.5%]); the AF for females and males due to high temperatures was 0.1% (95% CIs, 0.03%-0.3% and 0.1%-0.2%, respectively). Individuals of all marital statuses except single experienced substantial mortality fractions due to low temperatures (AFs, 1.7% [95% CI, 0.5%-2.8%] for married, 2.4% [95% CI, 0.7%-4.0%] for divorced, and 2.6% [95% CI, 1.4%-3.7%] for widowed), whereas all except married had significant mortality fractions due to high temperatures (AFs, 0.8% [95% CI, 0.1%-1.6%] for single, 0.3% [95% CI, 0.02%-0.5%] for divorced, and 0.1% [95% CI, 0.03%-0.2%] for widowed).

### Sensitivity Analyses

Our primary results were generally robust in the sensitivity analyses (eFigures 4-9 in Supplement 1). However, the association with low temperatures could extend as long as 20 days, such that our primary estimates on cold-related mortality burden were an underestimation (eFigures 4 and 5 in Supplement 1). Our estimation of associations with high temperatures was relatively conservative compared with the model using alternative knots for the temperature variable dimension (eFigure 6 in Supplement 1). The patterns of association with daily maximum or minimum temperatures were similar to that in the primary analysis: small discrepancies in the association at the cold and warm ends (eFigure 9 in Supplement 1) may be due to distinctive aspects of different exposure metrics.^23^

## Discussion

This case series assessed the association between daily temperature and mortality in the contiguous US from 2000 to 2020. We found that both low and high temperatures significantly increased mortality odds, with variation by cause of death and demographic factors, including age, sex, and marital status. The attributable death counts from high temperatures increased by more than 50% during the past 2 decades, whereas the counts from low temperatures increased by only 7%. We also observed regional differences in the attributable mortality fraction. Mortality due to endocrine, nutritional, and metabolic diseases, circulatory diseases, and respiratory diseases had significant AFs from low temperatures, and circulatory diseases and external causes had significant AFs from high temperatures. Low temperatures had higher overall AFs than high temperatures, particularly affecting older adults, females, and divorced or widowed individuals. For high temperatures, AFs were highest in younger people and single individuals.

Besides the estimates from the Centers for Disease Control and Prevention, previous temperature-attributable mortality estimates in the US were more than double our estimates, with similar ratios of the burden of cold vs heat. The GBD study based on nationwide death records in the US (1980-1988)^9^ found AFs of 3.63% (95% CI, 3.25%-3.95%) for low temperatures and 0.29% (95% CI, 0.19%-0.40%) for high temperatures. Using data from major US cities, MCC studies observed AFs of 5.51% (95% CI, 5.17%-5.82%) and 0.35% (95% CI, 0.30%-0.39%) for low and high temperatures, respectively (1985-2009)^14^ and excess annual deaths of 171 350 (95% CI, 148 863-196 266) and 20 064 (95% CI, 8703-35 204) for low and high temperatures, respectively, in North America (US and Canada; US data covered 1973-2006).^11^ A study based on 10 US cities (1985-2006)^10^ estimated that 12 000 (95% CI, 7400-16 500) annual deaths were attributable to high temperatures in the contiguous US; another study based on 297 populous counties (1997-2006)^24^ estimated an attributable mortality burden (excluding external causes) of 0.44% due to high temperatures. Differences among studies may be explained by spatiotemporal coverage of health data and methodological issues.^25^ Specifically, previous studies focused on major urban metropolitan areas, which may display different exposure-response patterns than suburban or rural areas, due to the urban heat island and differences in built environments, lifestyles, and sociodemographic profiles.^26,27^ We observed a higher cold AF but a lower heat AF in metropolitan vs nonmetropolitan areas, but with large uncertainties in these estimates. Existing evidence for the direction and magnitude of rural-suburban-urban differences regarding temperature-mortality associations remains inconclusive.^28^

Previous studies have found heterogeneous spatiotemporal patterns of attributable mortality burden across nations and regions,^11,12,13^ but little research has been conducted on subnational patterns. Both observational and projection studies have tended to find a decreasing low temperature– and an increasing high temperature–attributable mortality burden in the context of global warming.^11,29,30^ However, our study found an increasing trend for both low and high temperatures, with substantial year-to-year variation and a much greater increase for high temperatures. We note that in addition to a warming climate, growth and aging of the population can affect annual death counts from nonoptimal temperatures. The slightly increasing trend of cold-related mortality burden may be explained by aging,^12,15,31^ given our finding that older populations were more vulnerable to low temperatures than younger populations.

Research on the cause-specific mortality burden attributable to nonoptimal temperatures has been scarce. However, 1 US study^17^ found AFs of 7.15% (95% CI, 6.31%-7.85%) from low temperatures and 0.43% (95% CI, 0.37%-0.46%) from high temperatures for cardiorespiratory mortality (1987-2000). Regarding demographic heterogeneity, our findings are consistent with previous findings that age and sex are essential effect modifiers and that marital status may also play a role.^32^ Our findings of no associations with low temperatures for children and adolescents or young adults and increasing ORs for low temperatures with increasing age may be explained by decreasing cold-activated brown adipose tissue with increasing age.^33^ We note that statistical significance does not necessarily imply practical significance. For example, the statistically significant difference between sexes that we observed for the temperature-mortality association did not appear to be meaningfully different.

### Strengths and Limitations 

This study has several strengths. First, it was based on complete nationwide death records across 2 recent decades, ensuring robust and generalizable findings not limited to major cities. Second, our investigation into spatiotemporal patterns could help identify vulnerable areas and facilitate future public health policy and practice. Third, we evaluated associations of temperature with specific causes of death, providing evidence for tailoring cause-specific intervention guidelines. Fourth, taking advantage of individual-level records, we were able to evaluate the role of demographic factors on the individual level as opposed to the ecologic level, offering insights into vulnerable populations.

However, this study also has some limitations. First, we did not evaluate certain major causes of death (eg, renal diseases) or specific subcategories (eg, stroke as a subcategory of circulatory diseases) that are possibly related to nonoptimal temperatures.^22,23^ Second, our choice of a relatively short time window (a lag of 0-6 days) may have caused an underestimation of the low temperature mortality burden. Third, due to data use policy, we could not show the complete county-level attributable mortality burden to illustrate a comprehensive spatial pattern.

## Conclusions

In this case series, the findings underscore the association of both low and high temperatures with mortality in the contiguous US. The pattern of increasing nonoptimal temperature–related mortality over time, coupled with regional heterogeneity, emphasizes the urgent need for targeted public health interventions and improved public health preparedness. Even in the setting of global warming, low temperatures continued to account for most of the mortality burden, but the mortality burden from high temperatures exhibited a substantially greater increase during the study period. The findings also highlight differential vulnerabilities by cause of death and demographic factors, calling for tailored strategies in vulnerable populations.

## Notes

Supplement 1. **eTable 1.** Associations Between Temperature and All-Cause and Cause-Specific Mortality With the Respective Minimum Mortality Temperature (MMT) as the Reference **eTable 2.** Heterogeneity of Temperature-Mortality Associations by Age, Sex, and Marital Status **eTable 3.** Associations Between Temperature and All-Cause Mortality Stratified by Age, Sex, and Marital Status With the Respective Minimum Mortality Temperature (MMT) as the Reference **eTable 4.** Temporal Patterns of All-Cause Mortality Count Attributable to Low and High Temperatures **eTable 5.** All-Cause Mortality Attributable to Low and High Temperatures Stratified by Cause of Death, Age, Sex, and Marital Status **eFigure 1.** Spatial Patterns of Excess Average Annual All-Cause Mortality Rate Attributable to Low and High Temperatures **eFigure 2.** Spatial Patterns of Attributable Fractions For All-Cause Mortality Related to Low and High Temperatures on the State Level **eFigure 3.** Attributable All-Cause Mortality Fraction Stratified by County-Level Sociodemographic Characteristics and by Urbanicity **eFigure 4.** Sensitivity Analysis Varying the Time Window **eFigure 5.** Temporal Patterns of All-Cause Mortality Attributable to Low Temperatures in the Sensitivity Analysis Varying the Time Window **eFigure 6.** Sensitivity Analysis Varying the Knots for the Temperature Variable Dimension to County-Specific 25th, 50th, and 75th Percentiles **eFigure 7.** Sensitivity Analysis Additionally Controlling for Moving Averages of Daily Average PM_2.5_ Concentration Over Lag 0-6 Days (Specified as Linear) **eFigure 8.** Sensitivity Analysis Requiring Counties to Have ≥200 or ≥1000 Deaths To Be Included in the Metaregression Models **eFigure 9.** Sensitivity Analysis Using Daily Maximum or Minimum Temperature as the Primary Exposure Metric

Supplement 2.
Data Sharing Statement
