# Supplementary material for: Heat- and Cold-Related Mortality Burden in the US From 2000 to 2020
Source: JAMA Netw Open. 2025 Nov 7;8(11):e2542269. doi: 10.1001/jamanetworkopen.2025.42269 (PMC12595540; doi:10.1001/jamanetworkopen.2025.42269)
Supplement: Supplement 1. — eTable 1. Associations Between Temperature and All-Cause and Cause-Specific Mortality With the Respective Minimum Mortality Temperature (MMT) as the Reference eTable 2. Heterogeneity of Temperature-Mortality Associations by Age, Sex, and Marital Status eTable 3. Associations Between Temperature and All-Cause Mortality Stratified by Age, Sex, and Marital Status With the Respective Minimum Mortality Temperature (MMT) as the Reference eTable 4. Temporal Patterns of All-Cause Mortality Count Attributable to Low and High Temperatures eTable 5. All-Cause Mortality Attributable to Low and High Temperatures Stratified by Cause of Death, Age, Sex, and Marital Status eFigure 1. Spatial Patterns of Excess Average Annual All-Cause Mortality Rate Attributable to Low and High Temperatures eFigure 2. Spatial Patterns of Attributable Fractions For All-Cause Mortality Related to Low and High Temperatures on the State Level eFigure 3. Attributable All-Cause Mortality Fraction Stratified by County-Level Sociodemographic Characteristics and by Urbanicity eFigure 4. Sensitivity Analysis Varying the Time Window eFigure 5. Temporal Patterns of All-Cause Mortality Attributable to Low Temperatures in the Sensitivity Analysis Varying the Time Window eFigure 6. Sensitivity Analysis Varying the Knots for the Temperature Variable Dimension to County-Specific 25th, 50th, and 75th Percentiles eFigure 7. Sensitivity Analysis Additionally Controlling for Moving Averages of Daily Average PM2.5 Concentration Over Lag 0-6 Days (Specified as Linear) eFigure 8. Sensitivity Analysis Requiring Counties to Have ≥200 or ≥1000 Deaths To Be Included in the Metaregression Models eFigure 9. Sensitivity Analysis Using Daily Maximum or Minimum Temperature as the Primary Exposure Metric [file jamanetwopen-e2542269-s001.pdf]

Supplementary Online Content

Chu L, Dubrow R, Chen K. Heat- and cold-related mortality burden in the US from 2000 to 2020. *JAMA Netw Open.* 2025;8(11):e2542269. doi:10.1001/jamanetworkopen.2025.42269

eTable 1. Associations Between Temperature and All-Cause and Cause-Specific Mortality With the Respective Minimum Mortality Temperature (MMT) as the Reference

eTable 2. Heterogeneity of Temperature-Mortality Associations by Age, Sex, and Marital Status

eTable 3. Associations Between Temperature and All-Cause Mortality Stratified by Age, Sex, and Marital Status With the Respective Minimum Mortality Temperature (MMT) as the Reference

eTable 4. Temporal Patterns of All-Cause Mortality Count Attributable to Low and High Temperatures

eTable 5. All-Cause Mortality Attributable to Low and High Temperatures Stratified by Cause of Death, Age, Sex, and Marital Status

eFigure 1. Spatial Patterns of Excess Average Annual All-Cause Mortality Rate Attributable to Low and High Temperatures

eFigure 2. Spatial Patterns of Attributable Fractions For All-Cause Mortality Related to Low and High Temperatures on the State Level

eFigure 3. Attributable All-Cause Mortality Fraction Stratified by County-Level Sociodemographic Characteristics and by Urbanicity

eFigure 4. Sensitivity Analysis Varying the Time Window

eFigure 5. Temporal Patterns of All-Cause Mortality Attributable to Low Temperatures in the Sensitivity Analysis Varying the Time Window

eFigure 6. Sensitivity Analysis Varying the Knots for the Temperature Variable Dimension to County-Specific 25th, 50th, and 75th Percentiles

eFigure 7. Sensitivity Analysis Additionally Controlling for Moving Averages of Daily Average $PM_{2.5}$ Concentration Over Lag 0-6 Days (Specified as Linear)

eFigure 8. Sensitivity Analysis Requiring Counties to Have ≥200 or ≥1000 Deaths To Be Included in the Metaregression Models

eFigure 9. Sensitivity Analysis Using Daily Maximum or Minimum Temperature as the Primary Exposure Metric

This supplementary material has been provided by the authors to give readers additional information about their work.

© 2025 Chu L et al. *JAMA Network Open*.

eTable 1. Associations Between Temperature and All-Cause and Cause-Specific Mortality With the Respective Minimum Mortality Temperature (MMT) as the Reference

	MMT (percentile)	Odds ratio (95% confidence interval)			
		5th percentile	10th percentile	90th percentile	95th percentile
All-cause mortality	79.9	1.057 (1.051, 1.064)	1.047 (1.041, 1.053)	1.004 (1.002, 1.005)	1.011 (1.009, 1.013)
Endocrine, nutritional and metabolic diseases	86.8	1.137 (1.100, 1.176)	1.111 (1.077, 1.146)	1.001 (0.999, 1.003)	1.010 (1.002, 1.017)
Mental, behavioral and neurodevelopmental disorders	54.2	1.082 (1.054, 1.112)	1.061 (1.036, 1.086)	1.039 (1.017, 1.062)	1.056 (1.031, 1.080)
Nervous system diseases	59.7	1.066 (1.043, 1.090)	1.054 (1.033, 1.075)	1.022 (1.008, 1.038)	1.034 (1.017, 1.051)
Circulatory diseases	83.6	1.101 (1.090, 1.113)	1.083 (1.072, 1.093)	1.003 (1.002, 1.005)	1.014 (1.011, 1.017)
Respiratory diseases	83.7	1.105 (1.082, 1.129)	1.093 (1.072, 1.115)	1.003 (1.000, 1.005)	1.011 (1.005, 1.017)
Digestive diseases	86.8	1.040 (1.004, 1.077)	1.035 (1.002, 1.069)	1.001 (0.999, 1.004)	1.013 (1.004, 1.021)
External causes	4.8	1.000 (1.000, 1.000)	1.001 (0.995, 1.008)	1.127 (1.100, 1.154)	1.151 (1.123, 1.179)

© 2025 Chu L et al. *JAMA Network Open*.

eTable 2. Heterogeneity of Temperature-Mortality Associations by Age, Sex, and Marital Status

Potential effect modifier	p-value for the Q-statistic
Age	<0.0001
Sex	0.0015
Marital status	<0.0001

© 2025 Chu L et al. *JAMA Network Open*.

eTable 3. Associations Between Temperature and All-Cause Mortality Stratified by Age, Sex, and Marital Status With the Respective Minimum Mortality Temperature (MMT) as the Reference

	MMT (percentile)	Odds ratio (95% confidence interval)			
		5th percentile	10th percentile	90th percentile	95th percentile
Age (years)					
<5	5.9	1.000 (0.994, 1.006)	1.001 (0.985, 1.018)	1.048 (0.965, 1.138)	1.070 (0.983, 1.165)
5-24	1.0	1.080 (1.035, 1.128)	1.118 (1.052, 1.188)	1.303 (1.183, 1.436)	1.329 (1.205, 1.465)
25-44	28.1	1.018 (1.001, 1.034)	1.008 (0.998, 1.019)	1.052 (1.026, 1.078)	1.066 (1.040, 1.094)
45-64	77.9	1.056 (1.041, 1.071)	1.043 (1.030, 1.056)	1.006 (1.003, 1.009)	1.017 (1.012, 1.022)
65-74	84.4	1.074 (1.059, 1.090)	1.061 (1.047, 1.076)	1.002 (1.000, 1.004)	1.010 (1.006, 1.014)
75-84	83.4	1.063 (1.051, 1.075)	1.053 (1.042, 1.064)	1.002 (1.001, 1.004)	1.010 (1.006, 1.013)
>84	80.5	1.070 (1.058, 1.082)	1.057 (1.047, 1.068)	1.003 (1.001, 1.005)	1.010 (1.006, 1.013)
Sex					
Female	80.2	1.057 (1.048, 1.066)	1.049 (1.041, 1.056)	1.004 (1.002, 1.005)	1.011 (1.008, 1.014)
Male	80.3	1.057 (1.048, 1.066)	1.043 (1.035, 1.052)	1.004 (1.002, 1.005)	1.012 (1.009, 1.015)
Marital status					
Single	50.3	1.035 (1.021, 1.050)	1.025 (1.013, 1.037)	1.028 (1.016, 1.041)	1.046 (1.033, 1.060)
Married	82.0	1.051 (1.041, 1.062)	1.040 (1.031, 1.050)	1.001 (1.000, 1.002)	1.003 (1.000, 1.006)
Divorced	82.2	1.079 (1.061, 1.096)	1.061 (1.045, 1.077)	1.005 (1.002, 1.007)	1.018 (1.013, 1.023)
Widowed	82.3	1.070 (1.059, 1.081)	1.061 (1.051, 1.071)	1.003 (1.001, 1.005)	1.011 (1.008, 1.014)

© 2025 Chu L et al. *JAMA Network Open*.

eTable 4. Temporal Patterns of All-Cause Mortality Count Attributable to Low and High Temperatures

Year	Low temperature	High temperature
2000	45172 (28517, 61676)	2140 (890, 3390)
2001	42894 (26419, 59213)	2458 (1165, 3741)
2002	44259 (27531, 60831)	3467 (1789, 5157)
2003	46692 (29629, 63702)	2311 (1029, 3588)
2004	44096 (27792, 60404)	1480 (513, 2446)
2005	44958 (28254, 61411)	3499 (1718, 5283)
2006	39937 (23641, 56044)	3603 (1880, 5322)
2007	43510 (27443, 59461)	3260 (1585, 4936)
2008	46541 (29173, 63768)	2257 (972, 3542)
2009	44722 (27987, 61355)	2221 (993, 3447)
2010	46910 (30197, 63503)	4597 (2353, 6831)
2011	45922 (29033, 62730)	4655 (2353, 6940)
2012	39016 (22658, 55215)	4438 (2322, 6552)
2013	49378 (31319, 67311)	2896 (1415, 4377)
2014	49767 (31803, 67549)	2275 (925, 3619)
2015	47511 (30060, 64783)	3599 (1683, 5504)
2016	43883 (26603, 60994)	4550 (2242, 6847)
2017	44396 (26393, 62257)	3457 (1643, 5265)
2018	52074 (33426, 70579)	4512 (2253, 6761)
2019	50619 (31847, 69160)	4072 (1893, 6237)
2020	53579 (31700, 75289)	5944 (3035, 8842)
Average	45992 (28639, 63202)	3414 (1650, 5173)

© 2025 Chu L et al. *JAMA Network Open*.

eTable 5. All-Cause Mortality Attributable to Low and High Temperatures Stratified by Cause of Death, Age, Sex, and Marital Status

		Attributable fraction (%; 95% confidence interval)	
		Low temperature	High temperature
Cause of death			
	Endocrine, nutritional and metabolic diseases	5.5 (1.6, 9.2)	0.2 (-0.1, 0.5)
	Mental, behavioral and neurodevelopmental disorders	2.0 (-0.2, 4.1)	1.0 (-0.3, 2.3)
	Nervous system diseases	2.1 (-0.1, 4.2)	0.7 (0.0, 1.4)
	Circulatory diseases	3.5 (2.3, 4.7)	0.2 (0.1, 0.3)
	Respiratory diseases	4.6 (2.1, 7.0)	0.2 (0.0, 0.4)
	Digestive diseases	2.4 (-0.4, 5.1)	1.2 (-0.7, 3.0)
	External causes	0.0 (-0.1, 0.2)	6.0 (2.1, 9.8)
Age (years)			
	<5	0.6 (-1.7, 2.7)	3.8 (-6.3, 12.6)
	5-24	0.3 (-0.3, 0.9)	17.0 (5.2, 27.3)
	25-44	0.6 (-0.5, 1.7)	2.2 (-0.4, 4.6)
	45-64	1.7 (0.4, 2.9)	0.3 (-0.1, 0.7)
	65-74	2.7 (1.2, 4.3)	0.2 (0.0, 0.3)
	75-84	2.4 (1.1, 3.7)	0.1 (0.0, 0.2)
	>84	2.3 (1.1, 3.5)	0.1 (0.0, 0.3)
Sex			
	Female	2.0 (1.1, 2.8)	0.1 (0.0, 0.3)
	Male	1.6 (0.7, 2.5)	0.1 (0.1, 0.2)
Marital status			
	Single	1.1 (0.0, 2.1)	0.8 (0.1, 1.6)
	Married	1.7 (0.5, 2.8)	0.1 (0.0, 0.2)
	Divorced	2.4 (0.7, 4.0)	0.3 (0.0, 0.5)
	Widowed	2.6 (1.4, 3.7)	0.1 (0.0, 0.2)

© 2025 Chu L et al. *JAMA Network Open*.

eFigure 1. Spatial Patterns of Excess Average Annual All-Cause Mortality Rate Attributable to Low and High Temperatures

Complying with data use policy, counties with fewer than 10 attributable deaths were shown as missing (indicated by gray color). However, the contribution of these counties was taken into account in statistical analyses.

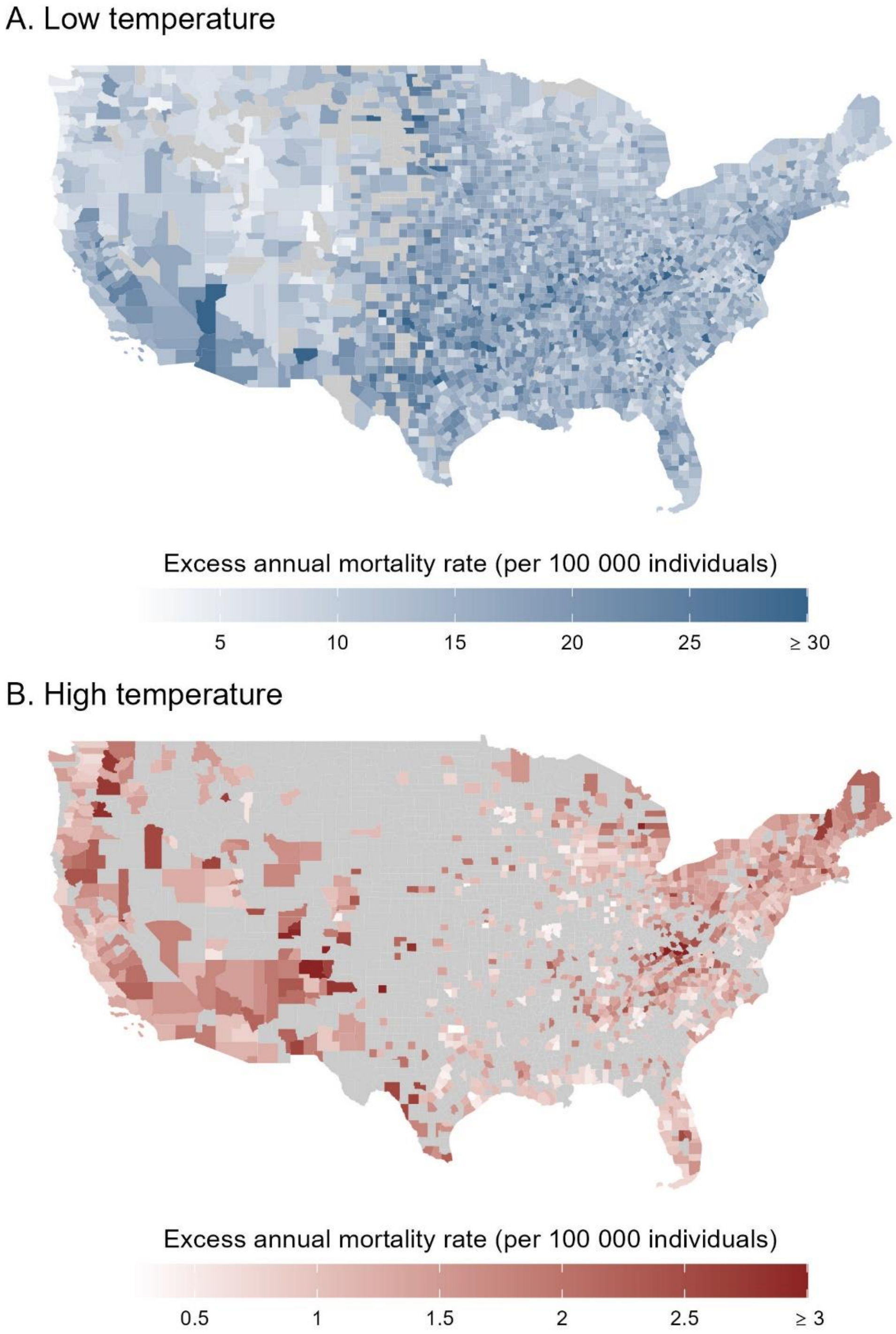

© 2025 Chu L et al. *JAMA Network Open*.

eFigure 2. Spatial Patterns of Attributable Fractions For All-Cause Mortality Related to Low and High Temperatures on the State Level

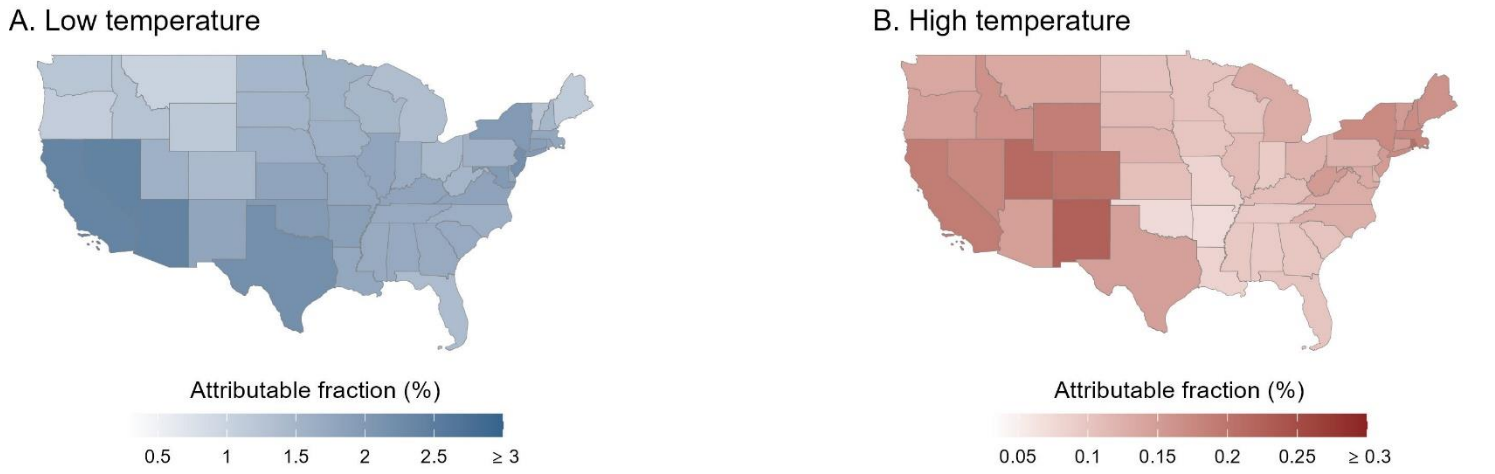

© 2025 Chu L et al. *JAMA Network Open*.

eFigure 3. Attributable All-Cause Mortality Fraction Stratified by County-Level Sociodemographic Characteristics and by Urbanicity

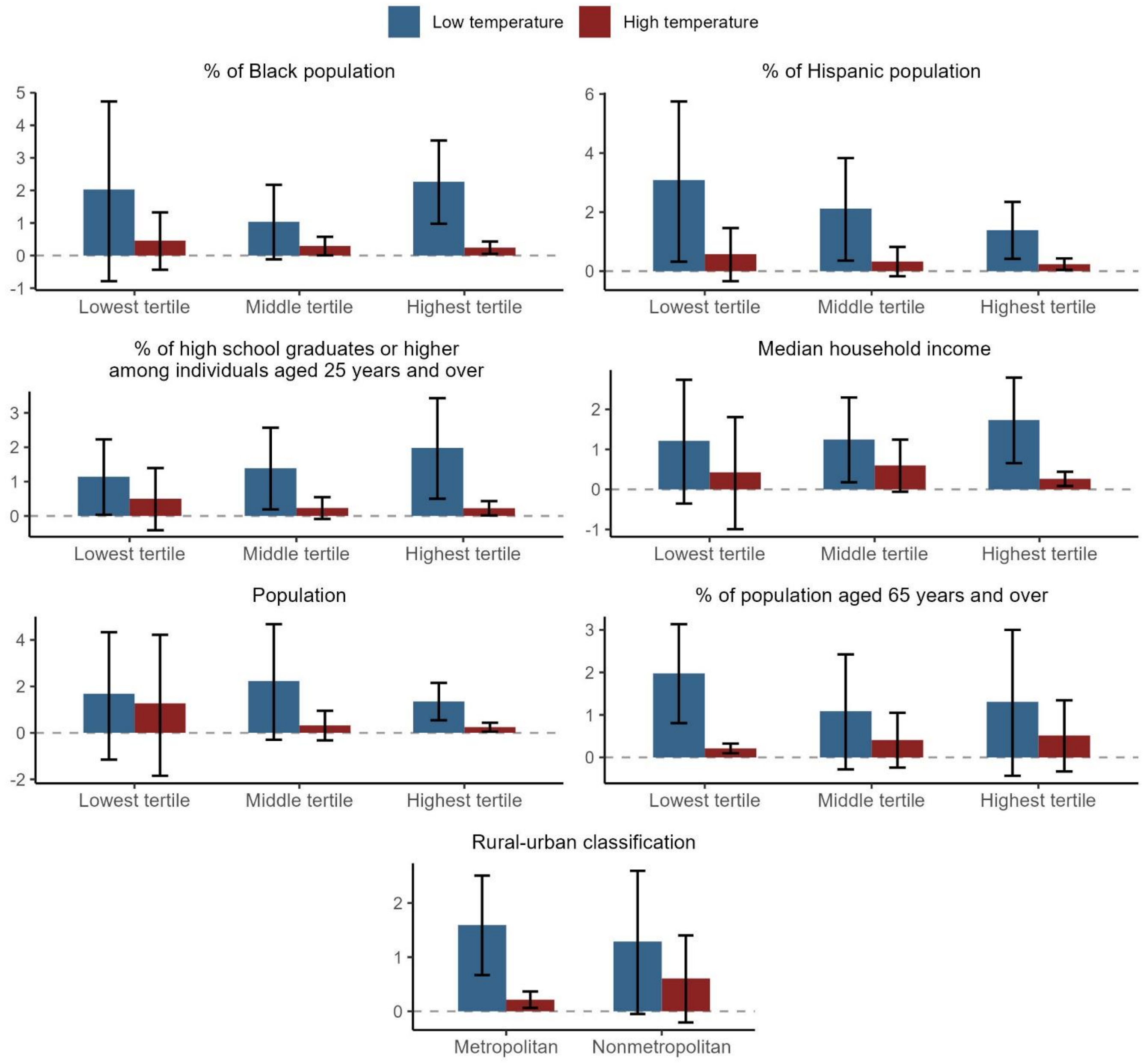

© 2025 Chu L et al. *JAMA Network Open*.

eFigure 4. Sensitivity Analysis Varying the Time Window

The respective minimum mortality temperature was used as the reference. The two vertical lines indicate the 1st and 99th percentiles of temperatures.

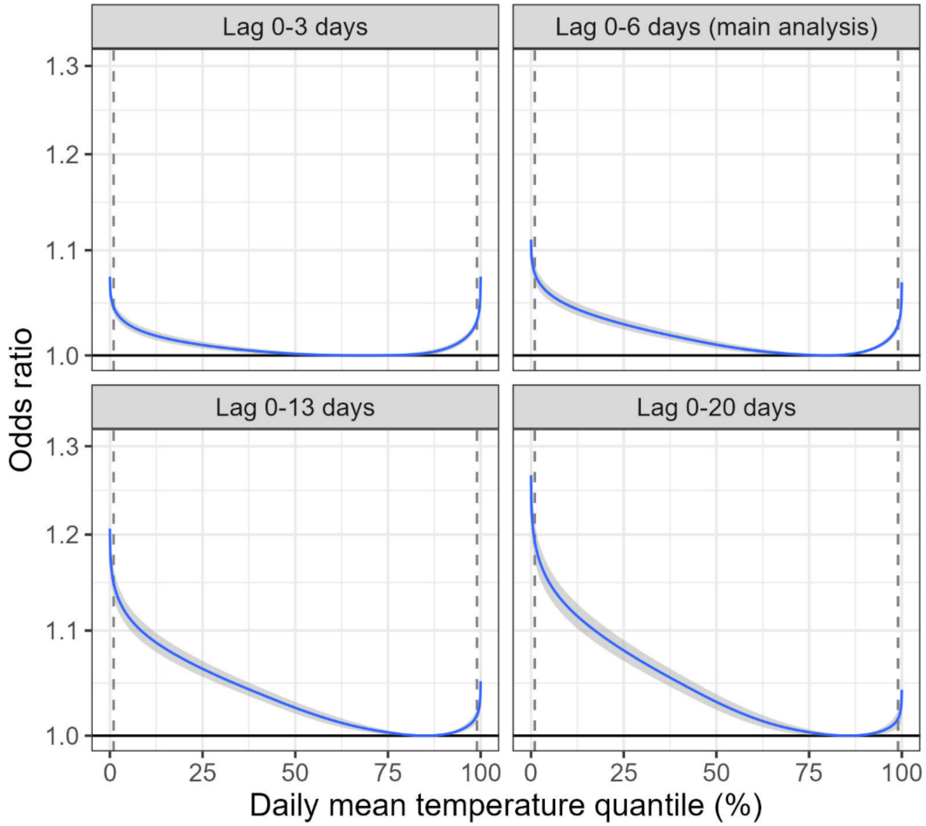

© 2025 Chu L et al. *JAMA Network Open*.

eFigure 5. Temporal Patterns of All-Cause Mortality Attributable to Low Temperatures in the Sensitivity Analysis Varying the Time Window

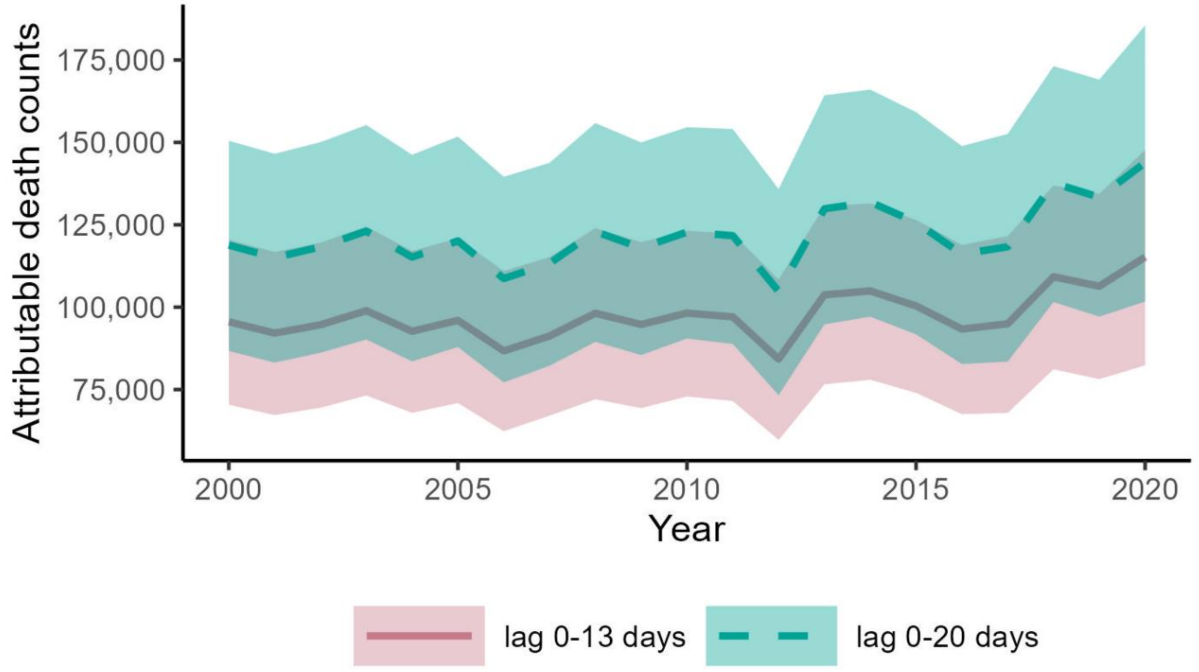

© 2025 Chu L et al. *JAMA Network Open*.

eFigure 6. Sensitivity Analysis Varying the Knots for the Temperature Variable Dimension to County-Specific 25th, 50th, and 75th Percentiles

The respective minimum mortality temperature was used as the reference. The two vertical lines indicate the 1st and 99th percentiles of temperatures.

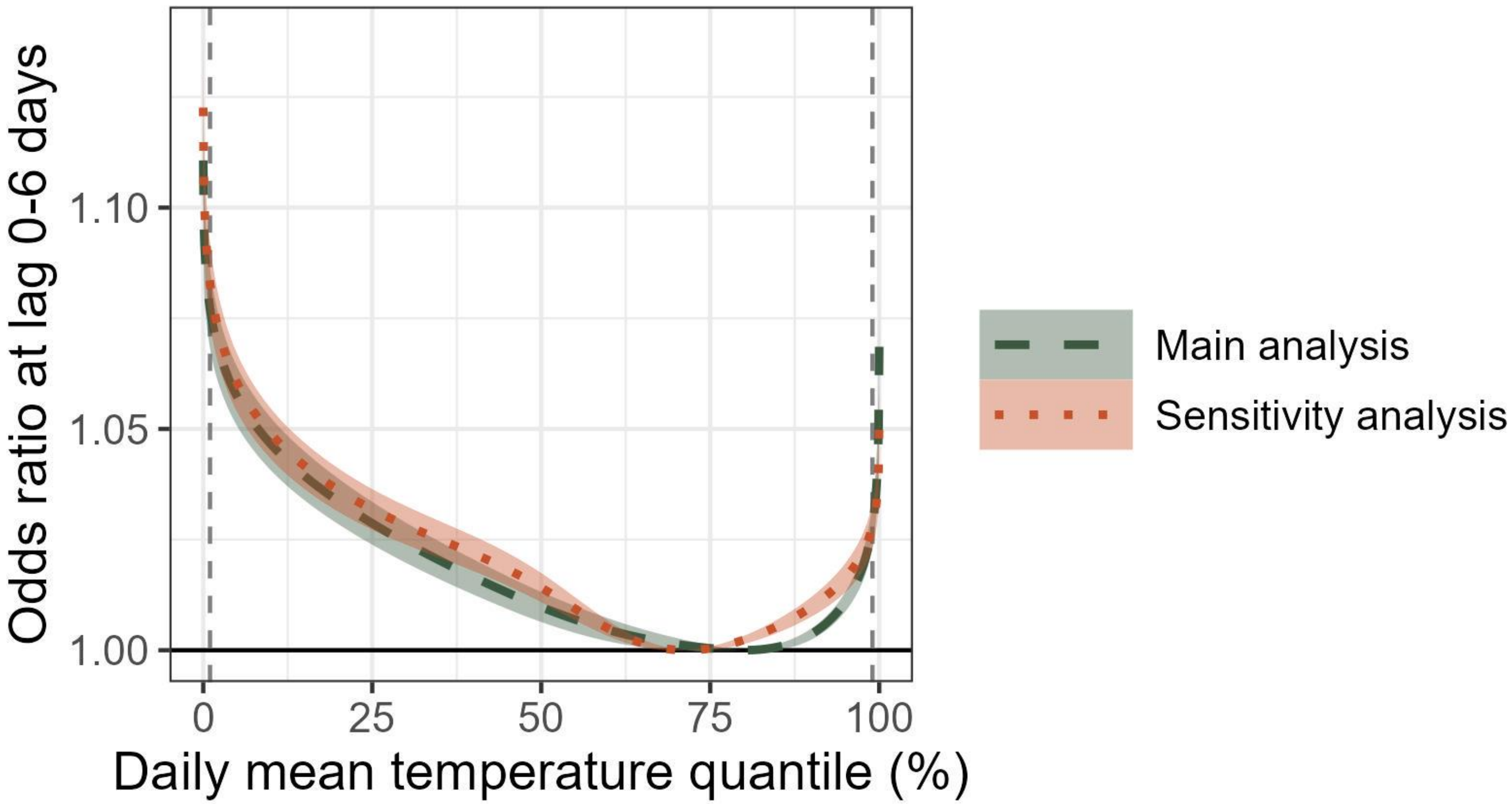

© 2025 Chu L et al. *JAMA Network Open*.

eFigure 7. Sensitivity Analysis Additionally Controlling for Moving Averages of Daily Average $PM_{2.5}$ Concentration Over Lag 0-6 Days (Specified as Linear)

The respective minimum mortality temperature was used as the reference. The two vertical lines indicate the 1st and 99th percentiles of temperatures.

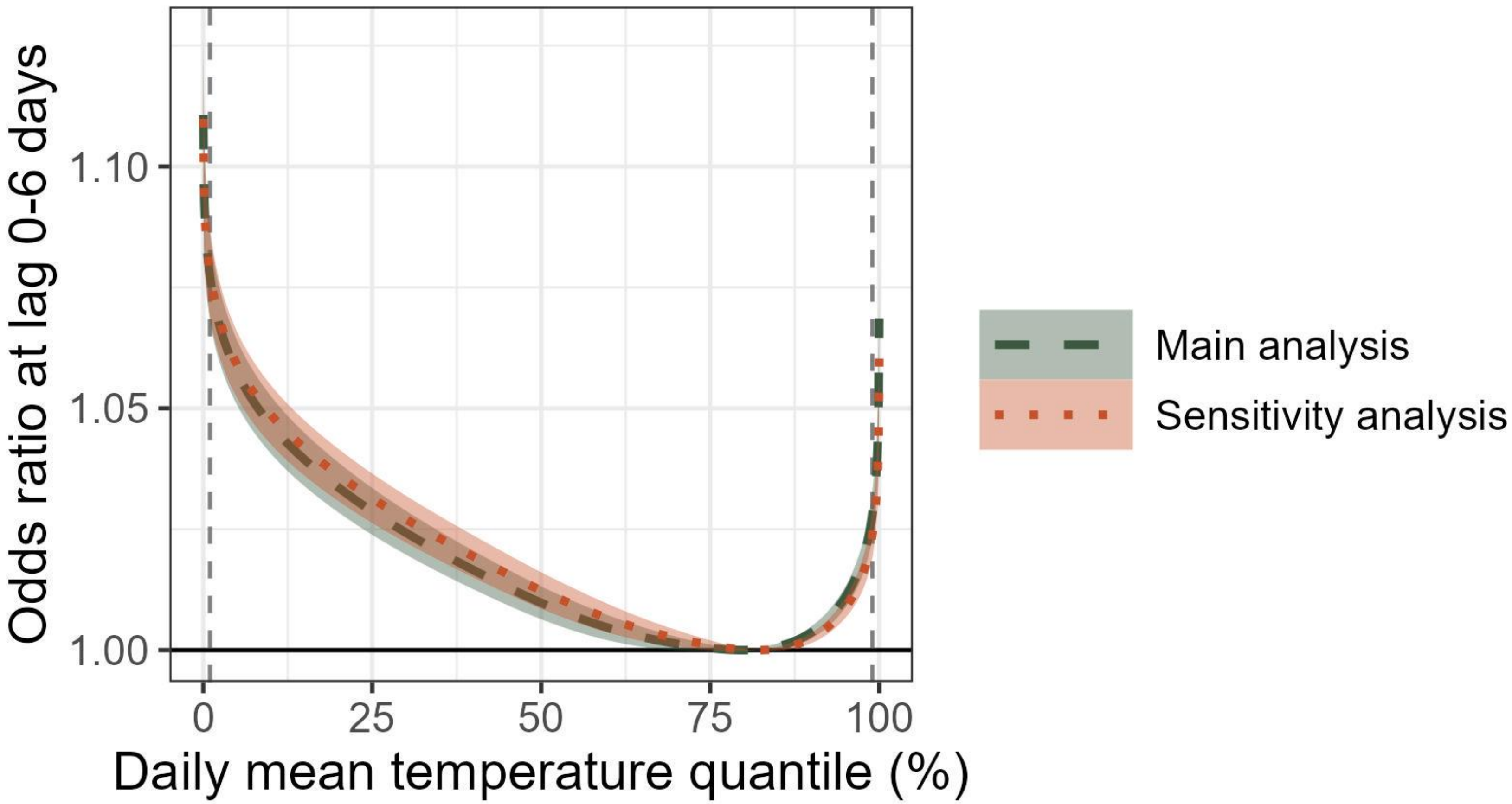

© 2025 Chu L et al. *JAMA Network Open*.

eFigure 8. Sensitivity Analysis Requiring Counties to Have ≥200 or ≥1000 Deaths To Be Included in the Metaregression Models

Stratified by cause of death, age, sex, and marital status. The respective minimum mortality temperature was used as the reference. The two vertical lines indicate the 1st and 99th percentiles of temperatures. Gray bands indicate the 95% confidence intervals for the main analysis (i.e., ≥500 deaths).

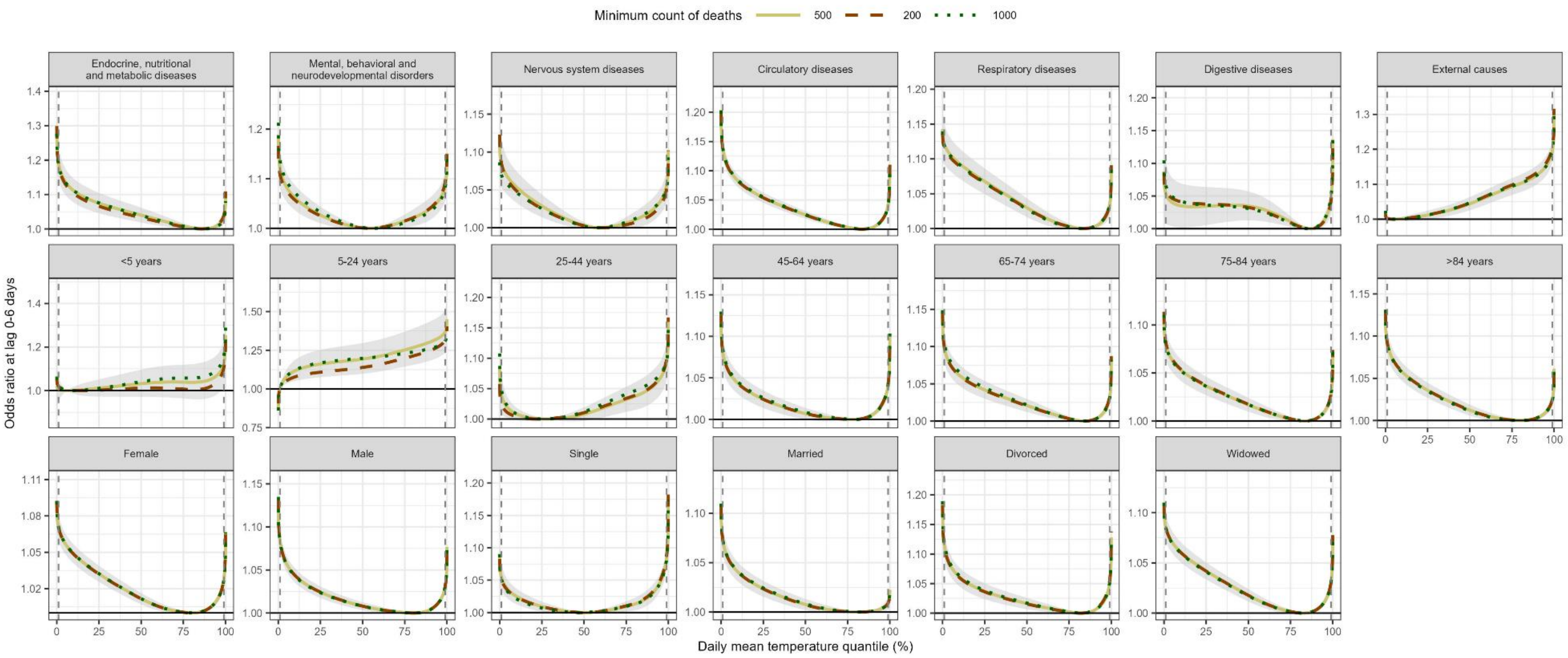

© 2025 Chu L et al. *JAMA Network Open*.

eFigure 9. Sensitivity analysis Using Daily Maximum or Minimum Temperature as the Primary Exposure Metric

The respective median temperature was used as the reference. The two vertical lines indicate the 1st and 99th percentiles of temperatures.

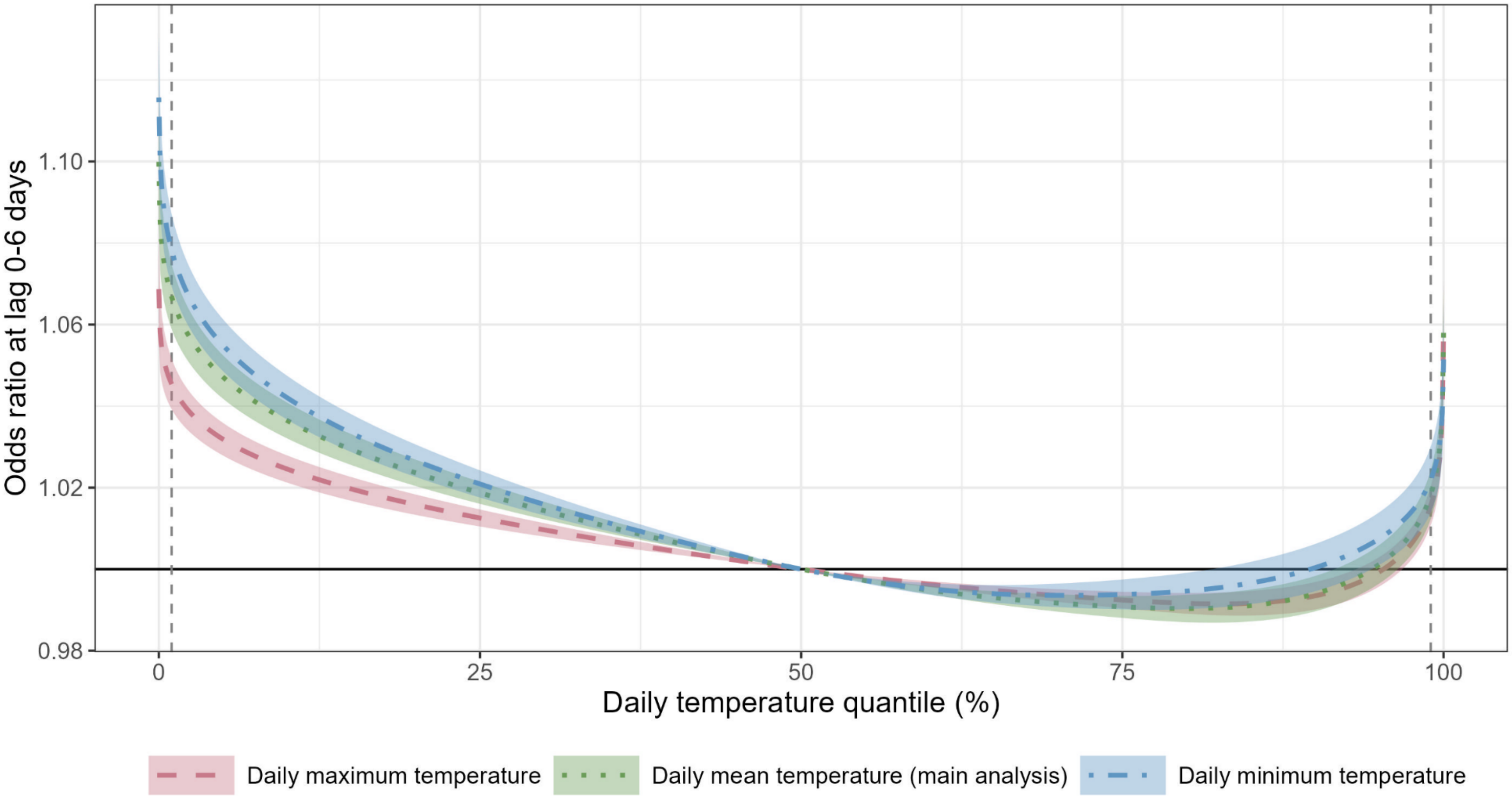

© 2025 Chu L et al. *JAMA Network Open*.
