# Supplementary material for: Heat- and Cold-Related Mortality Burden in the US From 2000 to 2020
Source: JAMA Netw Open. 2025 Nov 7;8(11):e2542269. doi: 10.1001/jamanetworkopen.2025.42269 (PMC12595540; doi:10.1001/jamanetworkopen.2025.42269)
Supplement: Supplement 2. — Data Sharing Statement [file jamanetwopen-e2542269-s002.pdf]

Data Sharing Statement

Chu. Heat- and Cold-Related Mortality Burden in the US From 2000 to 2020. *JAMA Netw Open*. Published November 07, 2025. doi:10.1001/jamanetworkopen.2025.42269

Data
Data available: No

Additional Information
Explanation for why data not available: The daily county-level mortality data cannot be shared per the data use agreement.
